# Operative burden and resuscitation resource utilisation in patients with traumatic shock requiring urgent surgical or endovascular intervention

**DOI:** 10.1007/s00068-026-03197-2

**Published:** 2026-04-27

**Authors:** Michael Noonan, Michael O’Loan, Simon Hendel, Mark Fitzgerald, Ee-Jun Ban, Frederick Huynh, Siobhan McKay, Adam Scorer, Gerard S. Goh, Charles H. C. Pilgrim

**Affiliations:** 1 National Trauma Research Institute, Melbourne, Australia; 2 Emergency & Trauma Centre, The Alfred Hospital, Melbourne, Australia; 3 Trauma Service, The Alfred Hospital, Melbourne, Australia; 4 School of Translational Medicine, Monash University, Melbourne, Australia; 5 School of Public Health and Preventative Medicine, Monash University, Melbourne, Australia; 6 Acute General Surgery Unit, The Alfred Hospital, Melbourne, Australia; 7 Hepatobiliary Surgery Unit, The Alfred Hospital, Melbourne, Australia; 8 Department of Anaesthesiology and Perioperative Medicine, The Alfred Hospital, Melbourne, Australia; 9 Department of Radiology, The Alfred Hospital, Melbourne, Australia; 10 C/o National Trauma Research Institute, Level 4/89 Commercial Rd, Melbourne, VIC 3004 Australia

**Keywords:** Injury, Trauma, Surgery, Shock, Bleeding, Massive transfusion

## Abstract

**Background:**

Patients with traumatic shock represent a high-risk subgroup within major trauma populations, yet the operative burden and resuscitation resource utilisation associated with urgent surgical and endovascular intervention in trauma systems managing predominantly blunt injury remain incompletely described.

**Aims:**

To describe the epidemiology, resuscitation resource utilisation, and operative burden of patients presenting with traumatic shock who require urgent surgical or endovascular intervention.

**Methods:**

We conducted a retrospective observational study at an Australian level 1 trauma centre using prospectively maintained trauma registries. Adult major trauma patients (≥ 16 years, Injury Severity Score ≥ 13) meeting institutional shocked trauma activation criteria between December 2022 and December 2024 were included. Resource utilisation, blood product transfusion, operative and endovascular interventions, procedural timing, and critical care outcomes were described. Comparisons were performed between shocked patients requiring urgent surgical or endovascular intervention and those managed without urgent procedures.

**Results:**

Of 3667 major trauma patients, 324 (8.8%) met shocked trauma criteria, and 138 (42.6% of shocked patients) underwent urgent surgical or endovascular intervention. These patients demonstrated substantial operative complexity; 37.7% required combined multispecialty interventions with 74.6% undergoing two or more operations within the first seven days. Median time to index urgent intervention was 150 min, and two-thirds of urgent surgical procedures occurred out-of-hours. ICU admission was near universal (94.9%) among patients requiring urgent intervention, with longer ICU LOS (median 8.0 days). Although median transfusion volumes were modest, marked heterogeneity in blood product utilisation was observed. High-volume transfusion (≥ 10 units PRBC within 24 h) occurred more frequently among patients requiring urgent intervention compared with those managed without urgent procedures (25.4% vs. 8.1%, *p* < 0.001).

**Conclusions:**

In a trauma system where blunt polytrauma predominates, patients with traumatic shock requiring urgent surgical or endovascular intervention represent a small but disproportionately resource-intensive cohort. Care is frequently iterative, multispecialty, and delivered out-of-hours, with substantial transfusion and critical care requirements. These findings highlight the need for sustained 24/7 trauma system capability and may inform future strategies for early risk stratification and resource planning in Australian trauma centres.

**Supplementary Information:**

The online version contains supplementary material available at 10.1007/s00068-026-03197-2.

## Introduction

Traumatic shock remains a leading cause of early mortality following major injury, with haemorrhage responsible for a substantial proportion of deaths within the first hours after trauma [1–3]. Despite advances in trauma systems and resuscitation practice, uncontrolled haemorrhage continues to drive early physiological collapse and necessitates urgent escalation of care, including operative or endovascular intervention.

Contemporary trauma resuscitation strategies, commonly framed under the principles of damage-control resuscitation (DCR), emphasise early haemorrhage control, permissive hypotension, minimisation of crystalloid administration, and early balanced blood product transfusion [4–6]. These approaches have been associated with improved survival in severely injured patients; however, they are resource intensive, requiring rapid mobilisation of blood products, operating theatres, specialist surgical teams, and critical care capacity.

Urgent surgical or endovascular intervention represents a pivotal point in the trajectory of patients with traumatic shock, particularly those with truncal or complex polytrauma. Delays to definitive haemorrhage control have consistently been associated with increased mortality [7–9]. While the clinical importance of timely operative intervention is well recognised, less attention has been directed toward the operational and resource implications of this subgroup, including transfusion burden, multispecialty operative involvement, timing of care delivery, and downstream critical care utilisation.

In non-trauma surgical populations, emergent operations are associated with substantially greater hospital resource utilisation compared with elective procedures, including longer length of stay, increased complications, and greater cost [10]. Within trauma systems this burden is further compounded by heterogeneity in injury patterns and physiological derangement, particularly in blunt polytrauma–dominant settings such as Australia and New Zealand [11]. Early care for shocked trauma patients also frequently occurs out-of-hours, placing additional strain on workforce availability, operating theatre access, and blood bank resources.

Despite the centrality of these patients to trauma system performance, there is limited literature describing resource utilisation specifically among patients presenting in traumatic shock who require urgent operative or endovascular intervention, particularly within Australasian trauma systems. Existing studies have largely focused on mortality and morbidity outcomes, with less emphasis on the parallel resource demands that underpin system capacity, resilience, and sustainability.

This study aims to describe the operative burden and resource utilisation associated with traumatic shock requiring urgent surgical or endovascular intervention at a level 1 trauma centre in Australia. Specifically, we characterise transfusion volume and timing, patterns of operative and endovascular care, multispecialty involvement, procedural timing, and critical care utilisation. By quantifying these domains, this work seeks to inform trauma system planning, benchmarking, and future quality-improvement initiatives in blunt polytrauma–dominant settings.

## Methods

### Study design and setting

A retrospective observational study was conducted at The Alfred Hospital, Melbourne, an adult level 1 trauma centre and the state’s largest trauma referral institution within the Victorian State Trauma System (VSTS). The study utilised data from the Alfred Health Trauma Registry (AHTR) and Alfred Trauma Resuscitation Registry (ATRR), both prospectively maintained institutional databases capturing all major trauma admissions and trauma team activations. The study period spans December 2022 to December 2024.

### Study population

Eligible patients included all adult major trauma cases (≥ 16 years) as defined by the VSTS (Injury Severity Score [ISS] ≥ 13) who met institutional “shocked trauma” activation criteria upon arrival to the Alfred Emergency and Trauma Centre. These criteria are based on prehospital or arrival physiology and include any of the following:

- Systolic blood pressure ≤ 90 mmHg,
- Shock index (heart rate/systolic blood pressure) > 1, or
- Administration of prehospital blood products.

Patients transferred secondarily from other hospitals after initial stabilisation, and those not meeting the shocked trauma activation threshold, were excluded.

### Data collection

Data were extracted from both registries and cross-linked using unique patient identifiers. Demographic, injury, and outcome variables included age, sex, mechanism of injury, Injury Severity Score (ISS), hospital and intensive care unit (ICU) length of stay (LOS), and in-hospital mortality. Baseline characteristics and outcomes are reported for shocked patients stratified by the requirement for urgent surgical or endovascular intervention.

### Resource utilisation and resuscitation

Resource utilisation data were collected across three predefined time intervals:

- Prehospital phase – administration of blood products prior to hospital arrival;
- Early hospital phase (0–5 h) – blood product administration, activation of major resources (operating theatre, anaesthesia, interventional radiology), and early operative or endovascular procedures;
- Early cumulative phase (0–24 h) – total transfusion volumes, ongoing operative or endovascular intervention, and ICU admission.

Blood product utilisation was quantified as cumulative units of packed red blood cells (PRBCs), fresh frozen plasma (FFP), platelets, and cryoprecipitate at each interval.

### Operative, endovascular and resuscitative procedures

Urgent operative or endovascular intervention was defined as direct transfer from the emergency department for emergent surgical or endovascular management. These procedures included interventions addressing both haemorrhagic and non-haemorrhagic life-threatening injuries occurring in patients meeting physiological criteria for traumatic shock. Procedures included:

- Decompressive cranial or spinal surgery;
- Thoracotomy or cardiothoracic surgical intervention;
- Trauma laparotomy or pelvic packing;
- External fixation for pelvic fractures;
- Fasciotomy or high-risk limb surgery (e.g. mangled extremity, bilateral femoral fractures);
- Initial major burns debridement; and
- Urgent endovascular procedures, including interventional radiology–led arterial embolisation and surgically led endovascular repair.

For each case, the lead surgical or interventional unit, involvement of multiple surgical or endovascular teams, and timing of the index procedure were recorded. Subsequent operative procedures within seven days of injury were also documented to characterise iterative or staged operative pathways.

Emergency department resuscitative procedures, including resuscitative thoracotomy, and in-hospital resuscitative adjuncts, such as unilateral or bilateral pleural decompression, were recorded for the entire shocked cohort and analysed separately from operative and endovascular procedures.

### Variables and definitions

- Polytrauma: Defined as an Abbreviated Injury Scale (AIS) score > 2 in at least two ISS regions [12, 13].
- Traumatic shock: Defined by the institutional shocked trauma activation criteria described above.
- Combined cases: Procedures requiring involvement of more than one surgical specialty during the index intervention.
- Massive transfusion: Defined as delivery of ≥ 5 units of PRBCs within the first 5 h of hospital care.
- ICU admission: Defined as ICU length of stay > 0 h.
- ICU LOS: Recorded in hours in the trauma registry and converted to days for reporting.

### Statistical analysis

Descriptive statistics were used to summarise patient demographics, injury severity, physiological parameters, procedural interventions, and resource utilisation. Categorical variables are presented as frequencies and percentages, and continuous variables as medians with interquartile ranges or means with standard deviations, as appropriate.

Comparisons between shocked patients requiring urgent intervention and those managed without urgent procedures were performed using χ² or Fisher’s exact tests for categorical variables and Wilcoxon rank-sum tests for continuous variables with non-normal distributions. Distributional differences in transfusion burden were further explored using graphical visualisation. Statistical significance was defined as a two-sided p value < 0.05. Analyses were performed using R (version 4.3.2; R Foundation for Statistical Computing, Vienna, Austria).

### Ethics

This study was approved by the Alfred Hospital Research Ethics Committee (Project No. 795/24).

## Results

### Study population

**Fig. 1:**
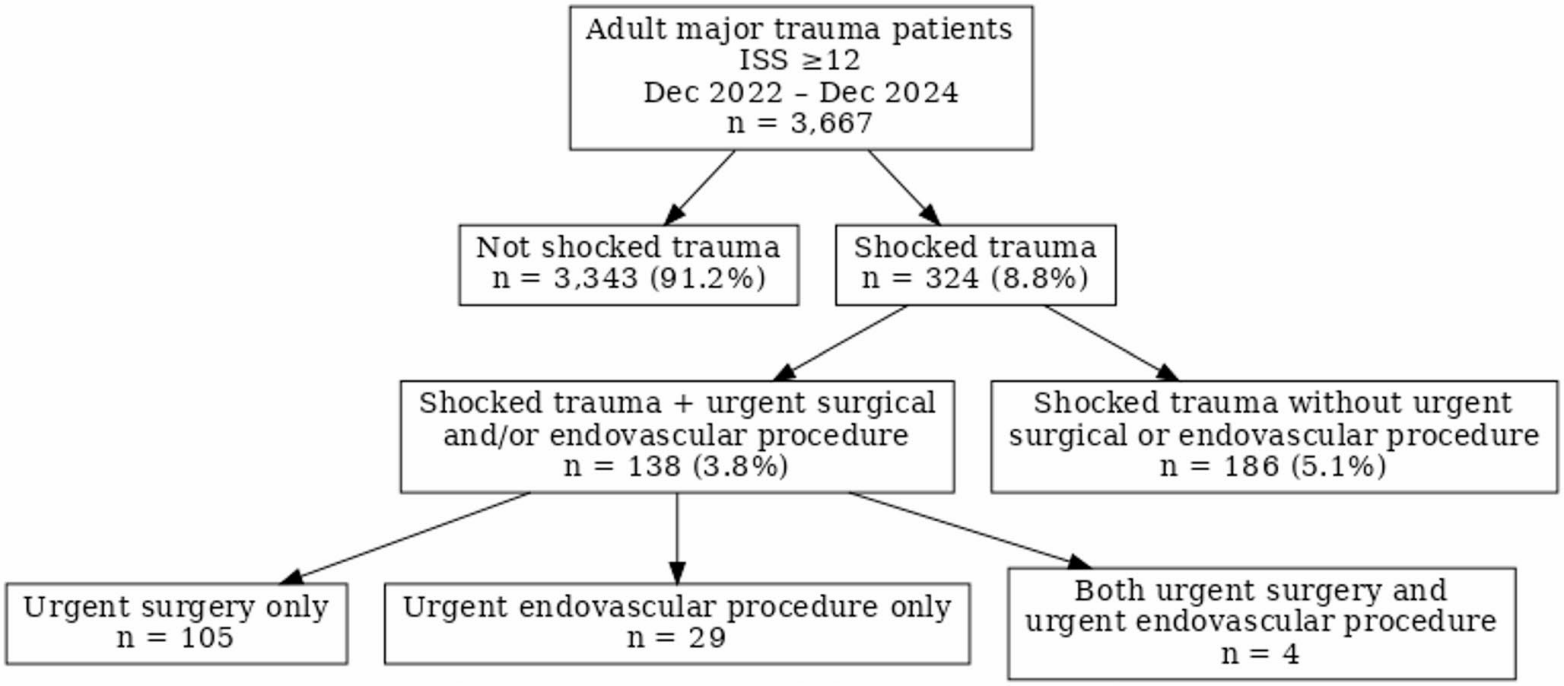
CONSORT flow diagram of major trauma patients by clinical shock status and urgent intervention (December 2022–December 2024)

Over the study period, 3,667 adult major trauma patients were identified, of whom 324 (8.8%) met clinical criteria for traumatic shock. Among shocked patients, 138 (42.6%) underwent an urgent surgical or endovascular intervention, representing 3.8% of the total major trauma cohort (Fig. 1). Polytrauma was present in 24.6% of the overall major trauma cohort, 63.6% of shocked patients, and 65.9% of those undergoing urgent intervention.

Within the shocked cohort, patients requiring urgent surgical or endovascular intervention were younger and had similar injury severity compared with those managed without urgent intervention (Table 1).

**Table 1:** Baseline characteristics of major trauma^a^ patients with clinical signs of shock by urgent intervention status (December 2022–December 2024)

Variable	Shocked^b^ NO urgent intervention^c^	Shocked^b^ WITH urgent intervention^c^
**Cases**		
Total (n (%))	186 (57.4)	138 (42.6)
Age (years) (mean (SD))	49.0 (21.1)	43.0 (19.3)
**Sex (n (%))**		
Male	139 (74.7)	99 (71.7)
Female	47 (25.3)	39 (28.3)
**ISS (median (IQR))**	27 (20–40)	29 (20–41)
**Polytrauma** ^**d**^ **(n (%))**	114 (61.3)	91 (65.9)
**Hospital LOS (days) (median (IQR)**	13.9 (5.3–28.0)	18.0 (9.3–29.8)
**ICU admission (n (%))**	156 (83.9)	131 (94.9)
**ICU LOS (days) (median (IQR)**	5.9 (2.3–13.6)	8.0 (3.7–14.0)
**Deaths (n (%))**		
Death in ED^e^	22 (11.8)	N/a
Death during acute hospital admission	54 (29.0)	19 (13.8)
Total deaths during index admission	76 (40.9)	19 (13.8)
**Mode of Transport from Scene (n (%))**		
Road Ambulance	84 (45.2)	66 (47.8)
Helicopter Ambulance	63 (33.9)	51 (37.0)
Other	39 (21.0)	21 (15.2)
**Mechanism of Injury (n (%))**		
Blunt	168 (90.3)	113 (81.9)
Penetrating	18 (9.7)	24 (17.4)
Other (including burns)	0 (0.0)	1 (0.7)

^a^Major trauma (MT) defined as ISS ≥ 13 & Age ≥ 16

^b^Shock defined clinically as pre-hospital (ph) or emergency department (ED) arrival SBP ≤ 90 OR SI > 1 OR received ph blood products

^c^Urgent surgical or endovascular intervention defined as direct transfer from the ED for: decompressive cranial surgery, decompressive spinal surgery, thoracotomy, laparotomy (or pelvic packing), external fixation for pelvic fracture, high risk limb(s) surgery - bilateral femur fractures / mangled limb / fasciotomy for compartment syndrome, initial major burns debridement, urgent endovascular procedure (including interventional radiology led arterial embolisation and surgically led endovascular repairs)

^d^Polytrauma defined as Abbreviated Injury Scale (AIS) score > 2 in at least two ISS body regions

^e^ED deaths occurred prior to operative decision making

ISS = Injury severity score

ICU = Intensive care unit

SBP = Systolic blood pressure

SI = Shock index

### Critical care utilisation and outcomes

ICU admission was common among shocked patients and occurred more frequently in those requiring urgent intervention (94.9% vs. 83.9%) (Table 1). Among ICU-admitted patients, ICU length of stay was longer in those requiring urgent intervention, with a median ICU LOS of 8.0 days compared with 5.9 days among those managed without urgent intervention (Table 1).

In-hospital mortality differed between shocked patients with and without urgent intervention, with lower mortality observed among patients undergoing urgent surgical or endovascular intervention (13.8% vs. 29.0%). Notably, 22 deaths occurred in the emergency department prior to operative decision-making, all among patients who did not undergo urgent intervention (Table 1).

### Blood product and fluid resuscitation

Pre-hospital blood product administration occurred in approximately one-third of shocked patients, with similar rates among those who did and did not undergo urgent intervention. In-hospital blood product use was common, occurring in 85.2% of shocked patients and 93.5% of those requiring urgent intervention (Table 2).

**Table 2:** Blood product resuscitation characteristics major trauma^a^ patients with clinical signs of shock by urgent intervention status (December 2022–December 2024)

Variable	Shocked^b^ NO urgent intervention^c^ (*n* = 186)	Shocked^b^ WITH urgent intervention^c^ (*n* = 138)
**Massive transfusion protocol**		
Activated (n (%))	137 (73.7)	105 (76.1)
Delivered ≥5u PRBC (0–5 h) (n (%))	29 (15.6)	59 (42.8)
**Prehospital PRBC**		
Any (n (%))	59 (31.7)	48 (34.8)
Units (median (IQR))	1 (0–3)	1 (0–3)
**PRBC 0–5 h**		
Any (n (%))	125 (67.2)	116 (84.1)
Units (median (IQR))	2 (0–4)	4 (2–8)
**PRBC 0–24 h**		
Any (n (%))	136 (73.1)	118 (85.5)
Units (median (IQR))	2 (0–4)	6 (2–10)
≥10 units PRBC in 24 h (n (%))	15 (8.1)	35 (25.4)
**FFP**		
0–5 h any (n (%))	117 (62.9)	116 (84.1)
24 h any (n (%))	126 (67.7)	119 (86.2)
**PRBC and FFP 0–5 h**^**d**^ (n (%))	116 (62.4)	114 (82.6)
**PRBC: FFP ratio 0–24 h**^**e**^ (median (IQR))	1.0 (0.8–1.5)	1.2 (0.9–1.6)
**Platelets**		
0–5 h any (n (%))	75 (40.3)	86 (62.3)
24 h any (n (%))	84 (45.2)	93 (67.4)
**Cryoprecipitate**		
0–5 h any (n (%))	16 (8.6)	43 (31.2)
24 h any (n (%))	27 (14.5)	63 (45.7)

^a^Major trauma (MT) defined as ISS ≥ 13 & Age ≥ 16

^b^Shock defined clinically as pre-hospital (ph) or emergency department (ED) arrival SBP ≤ 90 OR SI > 1 OR received ph blood products

^c^Urgent surgical or endovascular intervention defined as direct transfer from the ED for: decompressive cranial surgery, decompressive spinal surgery, thoracotomy, laparotomy (or pelvic packing), external fixation for pelvic fracture, high risk limb(s) surgery - bilateral femur fractures / mangled limb / fasciotomy for compartment syndrome, initial major burns debridement, urgent endovascular procedure (including interventional radiology led arterial embolisation and surgically led endovascular repairs)

^d^Co-administration of PRBC and FFP within the first 5 h is used as a surrogate indicator of balanced transfusion practice

^e^PRBC: FFP ratio calculated using cumulative 24-hour transfusion volumes among patients receiving both products

PRBC = Packed red blood cells

FFP = Fresh frozen plasma

Median early PRBC transfusion volumes were modest, with a median of 2 units within the first 5 h among shocked patients without urgent intervention and 4 units among shocked patients requiring urgent intervention. However, a substantial minority received high-volume transfusion. Delivered massive transfusion (≥ 5 units PRBC within 5 h) occurred in 42.8% of shocked patients undergoing urgent intervention, and ≥ 10 units PRBC within 24 h occurred in 25.4% of this group, compared with 15.4% among shocked patients managed without urgent intervention (Table 2, Appendix Table A2). Median PRBC: FFP ratios approximated 1:1 in both groups, consistent with balanced transfusion practice during early resuscitation (Table 2).

### Transfusion burden and heterogeneity among shocked patients

Despite modest median transfusion requirements, marked heterogeneity in blood product utilisation was observed within the shocked cohort. Cumulative 24-hour PRBC transfusion volumes were significantly higher among shocked patients requiring urgent surgical or endovascular intervention compared with those managed without urgent procedures (Wilcoxon rank-sum test, *p* < 0.001).

High-volume transfusion (≥ 10 units PRBC within 24 h) occurred more frequently in the urgent intervention group (25.4% vs. 8.1%; odds ratio 3.87, Fisher’s exact test, *p* < 0.001). Visualisation of transfusion distributions demonstrated substantial overlap in early transfusion volumes between groups, with progressive divergence over time. High cumulative transfusion volumes at 24 h were predominantly observed among patients requiring urgent operative or endovascular intervention, consistent with more profound haemorrhage in this subset of shocked patients (Supplementary Figure S1).

### Index resuscitation, surgical and endovascular procedures

In-hospital resuscitative adjuncts were common across the shocked cohort, with 59/324 (18.2%) patients undergoing pleural decompression. Bilateral pleural decompression occurred more frequently than unilateral decompression (12.3% vs. 5.9%). Emergency department resuscitative thoracotomy was performed in 6/324 (1.9%) shocked patients (Table 3).

**Table 3:** Index resuscitative, surgical and endovascular interventions in shocked^b^ major trauma^a^ patients undergoing urgent intervention^c^*

Procedure	*n*	%
**In-hospital resuscitative adjuncts (entire shocked cohort, ** ***n*** ** = 324)**		
Pleural decompression^d^		
Any Unilateral Bilateral	59 19 40	18.2 5.9 12.3
**Emergency department resuscitative intervention (entire shocked cohort, ** ***n*** ** = 324)**		
Resuscitative thoracotomy^e^	6	1.9
**Surgical and endovascular interventions (patients undergoing urgent intervention, ** ***n*** ** = 138)**		
***Surgical procedures***		
Laparotomy	32	23.2
Craniotomy / craniectomy	11	8.0
Sternotomy / thoracotomy	10	7.2
Wound washout and debridement	8	5.8
ICP monitor / EVD	6	4.3
External or internal fixation (femur)	6	4.3
External or internal fixation (tibia)	4	2.9
Cervical discectomy and/or fusion	4	2.9
External or internal fixation (upper limb)	3	2.2
Laparoscopy	2	1.4
Amputation	2	1.4
Thoracolumbar fusion	2	1.4
Femoral-pedal vascular bypass	1	0.7
***Endovascular procedures***		
Pelvic arterial embolisation	12	8.7
Splenic arterial embolisation	5	3.6
Thoraco-lumbar arterial embolisation	1	0.7
Upper limb arterial embolisation	1	0.7
TEVAR	1	0.7
EVAR (aortic bifurcation)	1	0.7

*Percentages are calculated using the urgent intervention cohort as the denominator. Patients may appear in multiple categories where both surgical and endovascular procedures were performed

^a^Major trauma (MT) defined as ISS ≥ 13 & Age ≥ 16

^b^Shock defined clinically as pre-hospital (ph) or emergency department (ED) arrival SBP ≤ 90 OR SI > 1 OR received ph blood products

^c^Urgent surgical or endovascular intervention defined as direct transfer from the ED for: decompressive cranial surgery, decompressive spinal surgery, thoracotomy, laparotomy (or pelvic packing), external fixation for pelvic fracture, high risk limb(s) surgery - bilateral femur fractures / mangled limb / fasciotomy for compartment syndrome, initial major burns debridement, urgent endovascular procedure (including interventional radiology led arterial embolisation and surgically led endovascular repairs)

^d^Pleural decompression refers to in-hospital finger thoracostomy performed as a resuscitative adjunct and is reported separately from operative procedures

^e^Resuscitative thoracotomy refers to emergency department–performed thoracotomy for haemorrhage control or cardiac tamponade prior to transfer to the operating theatre

ED = Emergency department

ICP = Intracranial pressure

EVD = External ventricular drain

TEVAR = Thoracic endovascular aortic repair

EVAR = Endovascular aneurysm repair

Among patients undergoing urgent intervention (*n* = 138), laparotomy was the most common index surgical procedure (23.2%), followed by neurosurgical and thoracic procedures. Endovascular interventions were less frequent and predominantly involved pelvic and splenic arterial embolisation. Patients undergoing both surgical and endovascular procedures were included in multiple categories as appropriate (Table 3).

### Operative pathways and timing

Combined urgent interventions involving more than one surgical or endovascular specialty occurred in 37.7% of patients requiring urgent intervention. Multiple operative episodes were common, with 74.6% of patients undergoing urgent intervention requiring two or more procedures within the first 7 days (Table 4).

**Table 4:** Operative pathways and timing in shocked^b^ major trauma^a^ patients undergoing urgent intervention^c^ (patient-level, *n* = 138)^+^

Variable	Value
Combined urgent intervention cases^d^ (n (%))	52 (37.7)
Patients with ≥ 2 interventions within first 7 days (n (%))	103 (74.6)
Time to index urgent intervention (min) - median (IQR)	150 (91–230)
**Timing of index urgent surgical procedure (** ***n*** ** = 109)**	
Weekday in-hours (08:00–16:59) (n (%))	24 (22.0)
Weekday out-of-hours (17:00–07:59) (n (%))	51 (46.8)
Weekend in-hours (08:00–16:59) (n (%))	13 (11.9)
Weekend out-of-hours (17:00–07:59) (n (%))	21 (19.3)

^+^Percentages calculated using the urgent intervention cohort as the denominator unless otherwise specified

^a^Major trauma (MT) defined as ISS ≥ 13 & Age ≥ 16

^b^Shock defined clinically as pre-hospital (ph) or emergency department (ED) arrival SBP ≤ 90 OR SI > 1 OR received ph blood products

^c^Urgent surgical or endovascular intervention defined as direct transfer from the ED for: decompressive cranial surgery, decompressive spinal surgery, thoracotomy, laparotomy (or pelvic packing), external fixation for pelvic fracture, high risk limb(s) surgery - bilateral femur fractures / mangled limb / fasciotomy for compartment syndrome, initial major burns debridement, urgent endovascular procedure (including interventional radiology led arterial embolisation and surgically led endovascular repairs)

^d^Combined intervention cases are defined as those requiring more than one specialty involvement during the index procedure

The median time from hospital arrival to commencement of the index urgent procedure was 150 min (IQR 91–230). Among patients undergoing urgent surgery (*n* = 109), only 22.0% of procedures commenced during weekday in-hours. The majority occurred out-of-hours (66.1%), and 31.2% occurred on weekends (Table 4).

### Operative burden by speciality

A total of 518 procedures were performed within the first 7 days among patients undergoing urgent intervention. Orthopaedic surgery accounted for the greatest procedural burden (46.4% of all procedures), followed by plastic surgery (18.2%) and general surgery (14.1%). Procedural intensity varied by specialty, with orthopaedics and burns surgery demonstrating higher mean numbers of procedures per involved patient (Table 5).

**Table 5:** Operative burden by surgical specialty within the first 7 days among shocked^b^ major trauma^a^ patients undergoing urgent intervention^c^ (procedure-level, *n* = 518)^

Surgical specialty	Patients involved (*n*)	Procedures (*n*)	% of all procedures	Mean procedures per patient†
Orthopaedics	67	240	46.4	3.6
Plastic Surgery	36	94	18.2	2.5
General Surgery	44	73	14.1	1.7
Neurosurgery	33	51	9.9	1.5
Cardiothoracic Surgery	20	26	5.0	1.3
Vascular Surgery	8	14	2.7	1.8
Burns Surgery	1	8	1.5	8.0
Urology	4	4	0.8	1.0
ENT	3	3	0.6	1.0
Ophthalmology	2	2	0.4	1.0
Gastroenterology	1	1	0.2	1.0
Maxillofacial Surgery	1	1	0.2	1.0

^Percentages calculated using the total number of procedures performed within the first 7 days as the denominator

†Mean procedures per patient calculated as total procedures divided by number of patients undergoing ≥ 1 procedure by that specialty within the first 7 days. Patients may contribute to multiple specialties

^a^Major trauma (MT) defined as ISS ≥ 13 & Age ≥ 16

^b^Shock defined clinically as pre-hospital (ph) or emergency department (ED) arrival SBP ≤ 90 OR SI > 1 OR received ph blood products

^c^Urgent surgical or endovascular intervention defined as direct transfer from the ED for: decompressive cranial surgery, decompressive spinal surgery, thoracotomy, laparotomy (or pelvic packing), external fixation for pelvic fracture, high risk limb(s) surgery - bilateral femur fractures / mangled limb / fasciotomy for compartment syndrome, initial major burns debridement, urgent endovascular procedure (including interventional radiology led arterial embolisation and surgically led endovascular repairs)

ENT = Ear, Nose and Throat

### Operative timing by specialty

Time to index urgent procedure varied by specialty, with earlier intervention observed for cardiothoracic and neurosurgical procedures and longer times for spinal surgery and interventional radiology cases (Appendix Table A1).

## Discussion

In this single-centre study from an Australian Level 1 trauma service, we describe the operative burden and resource utilisation associated with traumatic shock requiring urgent surgical or endovascular intervention. Among patients meeting institutional shocked trauma criteria, fewer than half progressed to urgent operative or endovascular management; however, those who did represent a distinct, high-resource subgroup characterised by substantial transfusion requirements, multispecialty operative involvement, and increased critical care utilisation.

The lower mortality observed among patients undergoing urgent intervention likely reflects survivor treatment bias. Patients must survive initial resuscitation and diagnostic evaluation to reach operative or endovascular intervention. In this cohort, 22 deaths occurred in the emergency department prior to operative decision-making and were confined to the non-intervention group (Table 1), suggesting that early non-survivable injury contributed substantially to the observed mortality difference.

Consistent with the epidemiology of trauma in Australia and New Zealand, the cohort was dominated by blunt force polytrauma [11], reflecting a high burden of multi-system injury in this population. Despite this, the need for urgent operative intervention was common and frequently complex. Laparotomy remained the most common index operative procedure, but neurosurgical, thoracic, orthopaedic, vascular, and endovascular interventions all contributed meaningfully, highlighting the breadth of subspecialty expertise required early in care. Over one-third of patients undergoing urgent intervention required combined procedures involving multiple surgical specialties, and nearly three-quarters underwent two or more operative episodes within the first seven days. These findings reinforce that early trauma surgery in shocked patients is rarely a single definitive event and more commonly reflects iterative, staged damage-control pathways [4–6].

Blood product utilisation demonstrated marked heterogeneity within the shocked cohort. While median transfusion volumes were modest, a substantial minority of patients—predominantly those requiring urgent operative or endovascular intervention—received large volumes of blood products. Importantly, early transfusion volumes overlapped between patients who did and did not proceed to urgent intervention, with divergence becoming apparent over the first 24 h. Median PRBC: FFP ratios approximated 1:1 across the cohort, suggesting adherence to contemporary balanced transfusion strategies within the institutional massive transfusion protocol. Together, these findings suggest divergent haemorrhage trajectories within shocked patients, with a subgroup exhibiting ongoing or refractory bleeding that drives disproportionate resource utilisation. This pattern has implications for early risk stratification and supports continued emphasis on dynamic reassessment rather than reliance on single time-point physiological markers.

The timing and context of operative care further emphasise the system-level burden imposed by this cohort. In blunt polytrauma–dominant systems, time to operative intervention frequently reflects the need for rapid diagnostic imaging, multidisciplinary coordination, and prioritisation of competing life-threatening injuries [7–9]. Most urgent surgical procedures occurred out-of-hours, with nearly one-third commencing on weekends and fewer than one-quarter occurring during weekday in-hours. This underscores the requirement for sustained 24/7 surgical, anaesthetic, radiology, and critical care capability in major trauma centres, and challenges models of care that rely heavily on daytime capacity. The high rates of ICU admission and prolonged ICU LOS among patients requiring urgent intervention further amplify the downstream resource implications of this group.

Taken together, these findings highlight that patients with traumatic shock who require urgent operative or endovascular intervention represent a small but disproportionately resource-intensive subgroup within major trauma populations. In blunt polytrauma–dominant systems such as those in Australia and New Zealand, planning for trauma care must account not only for case volume but also for the complexity, timing, and iterative nature of care required by shocked patients. Improved early identification of patients likely to follow high-resource haemorrhage trajectories may allow better alignment of surgical, transfusion, and critical care resources with patient need. These findings reinforce the importance of trauma systems capable of rapidly mobilising multidisciplinary surgical, anaesthetic, radiological, and transfusion resources for this critically injured cohort.

## Limitations

This was a retrospective, single-centre observational study, which may limit generalisability to other trauma systems. Analyses were based on registry data and predefined time windows and therefore lack precise event-level timestamps for transfusion and procedural interventions. The study was descriptive in nature and not designed to establish causal relationships or formally classify haemorrhage phenotypes. Despite these limitations, the findings provide a contemporary description of operative burden and resource utilisation among patients with traumatic shock in a high-volume Australian trauma centre. The study did not include detailed health economic analysis of operative or transfusion costs, which may be an important area for future investigation.

## Conclusions

Patients with traumatic shock who require urgent surgical or endovascular intervention represent a small but disproportionately resource-intensive subgroup within major trauma populations. In a predominantly blunt polytrauma setting, these patients frequently require multispecialty, iterative operative care, substantial blood product transfusion, and greater critical care utilisation, with much of this activity occurring out-of-hours. Marked heterogeneity in transfusion requirements was observed, with high-volume blood product utilisation concentrated among patients requiring urgent intervention. These findings highlight the importance of sustained 24/7 trauma system capability and may inform future strategies for early risk stratification and resource planning in Australian trauma centres.

## Supplementary Information

Below is the link to the electronic supplementary material.

Supplementary Material 1

Supplementary Material 2

## Data Availability

No datasets were generated or analysed during the current study.

## Declarations

### Use of generative AI

Generative artificial intelligence (AI) tools were used to assist with the organisation, refinement, and editing of manuscript text. All analyses, interpretations, and conclusions were conceived, verified, and approved by the authors, who take full responsibility for the content of the manuscript.
