# Supplementary material for: Operative burden and resuscitation resource utilisation in patients with traumatic shock requiring urgent surgical or endovascular intervention
Source: Eur J Trauma Emerg Surg. 2026 Apr 27;52(1):149. doi: 10.1007/s00068-026-03197-2 (PMC13121394; doi:10.1007/s00068-026-03197-2)
Supplement: Supplementary file 1 — Supplementary Material 1 [file 68_2026_3197_MOESM1_ESM.docx]

## Appendix. Table A1. Time to index urgent surgical or endovascular procedure stratified by specialty^#^

| **Lead specialty** | **Procedures (n)** | **Time to index procedure (mins), median (IQR)** |
| --- | --- | --- |
| General Surgery | 30 | 116 (60–165) |
| Interventional radiology | 28 | 220 (168–274) |
| Neurosurgery | 18 | 112 (88–134) |
| Orthopaedic Surgery | 18 | 156 (133–206) |
| Cardiothoracic Surgery | 15 | 91 (50–124) |
| Spinal Surgery | 11 | 320 (189–456) |
| Vascular Surgery | 11 | 151 (102–260) |
| Plastic Surgery | 4 | 118 (108–152) |
| Burns Surgery | 1 | 230 (230–230) |
| ENT Surgery | 1 | 190 (190–190) |

^#^Time calculated from hospital arrival to commencement of the index urgent surgical or endovascular procedure.

ENT = Ear, Nose and Throat

Appendix. Table A2. Additional blood product and fluid resuscitation characteristics major trauma^a^ patients with clinical signs of shock by urgent intervention status (December 2022–December 2024)

| **Variable** | **Shocked^b^ NO urgent intervention^c^** (n = 186) | **Shocked^b^ WITH urgent intervention^c^** (n = 138) |
| --- | --- | --- |
| Prehospital vasopressors – any (n (%)) | 50 (26.9) | 28 (20.3) |
| Prehospital crystalloid (ml) – any (n (%)) | 87 (46.8) | 67 (48.6) |
| Prehospital crystalloid (ml) – median (IQR) | 0 (0–700) | 0 (0–585) |
| ≥10u PRBC in 24h (n (%)) | 15 (8.1) | 35 (25.4) |
| Crystalloid 0-5h (ml) – median (IQR) | 200 (0–250) | 100 (0–200) |
| Crystalloid 24h (ml) – median (IQR) | 2200 (1000–3388) | 2600 (1992–3250) |

^a^Major trauma (MT) defined as ISS ≥13 & Age ≥16

^b^Shock defined clinically as pre-hospital (ph) or emergency department (ED) arrival SBP≤90 OR SI>1 OR received ph blood products

^c^Urgent surgical or endovascular intervention defined as direct transfer from the ED for: decompressive cranial surgery, decompressive spinal surgery, thoracotomy, laparotomy (or pelvic packing), external fixation for pelvic fracture, high risk limb(s) surgery - bilateral femur fractures / mangled limb / fasciotomy for compartment syndrome, initial major burns debridement, urgent endovascular procedure (including interventional radiology led arterial embolisation and surgically led endovascular repairs)

PRBC = Packed red blood cells
