# Supplementary material for: Operative burden and resuscitation resource utilisation in patients with traumatic shock requiring urgent surgical or endovascular intervention
Source: Eur J Trauma Emerg Surg. 2026 Apr 27;52(1):149. doi: 10.1007/s00068-026-03197-2 (PMC13121394; doi:10.1007/s00068-026-03197-2)
Supplement: Supplementary file 2 — Supplementary Material 2 [file 68_2026_3197_MOESM2_ESM.docx]

Supplementary Figure S1. Distribution of PRBC transfusion volumes in shocked trauma patients stratified by urgent intervention

1.
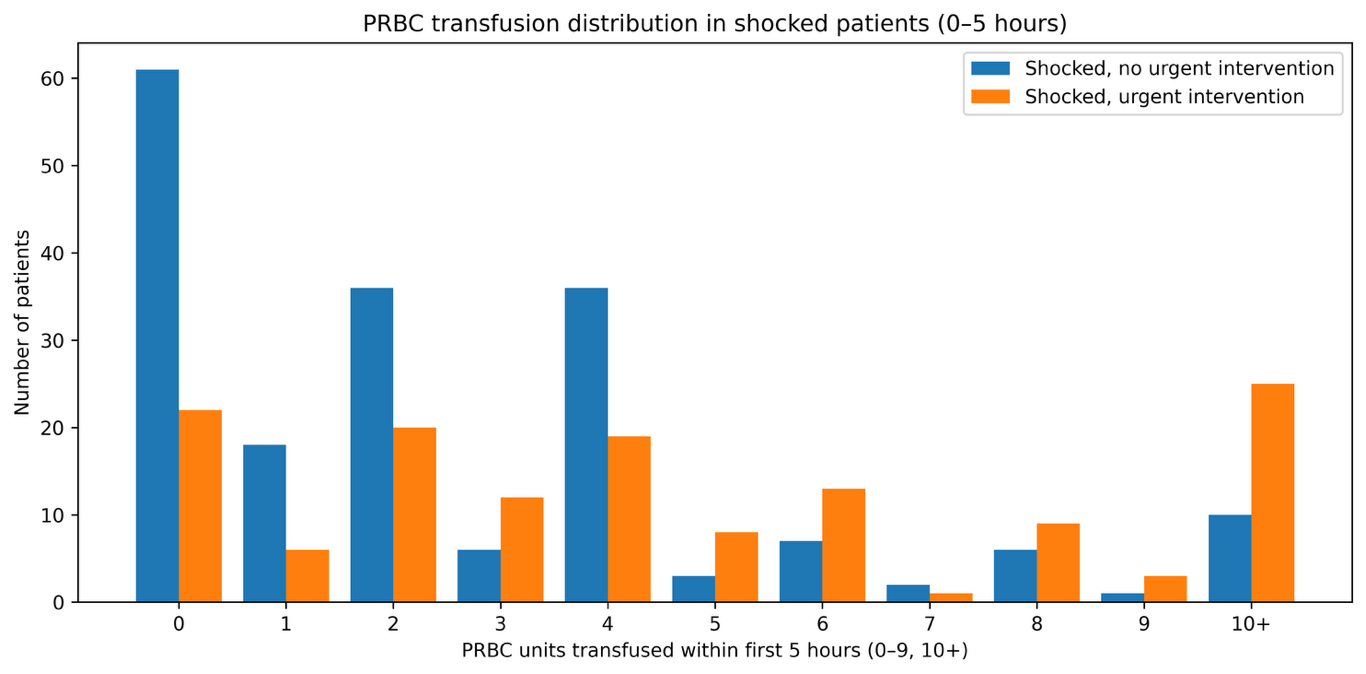


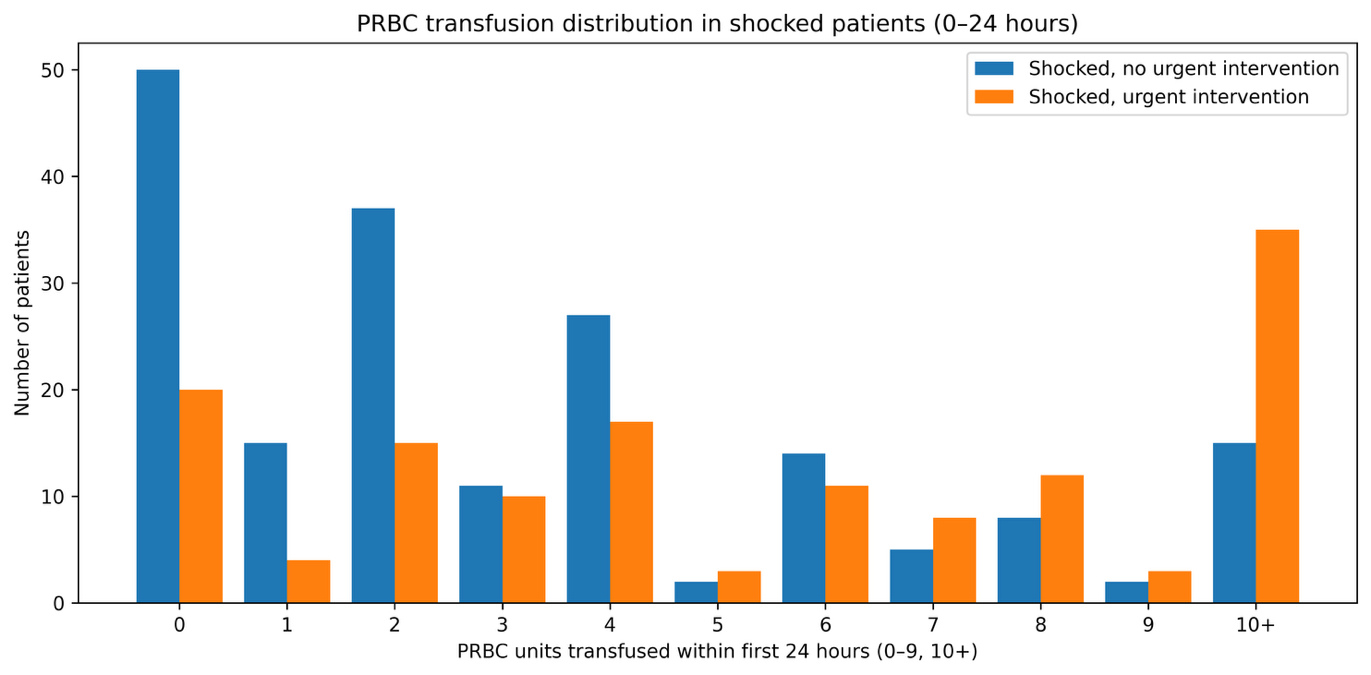


B.

Distribution of packed red blood cell (PRBC) transfusion volumes within the first 5 hours (A) and first 24 hours (B) after hospital arrival among shocked trauma patients, stratified by urgent surgical or endovascular intervention. PRBC volumes are displayed as discrete unit categories from 0 to 9, with all values ≥10 units grouped as “10+”, reflecting clinically relevant massive transfusion thresholds. Early transfusion volumes overlap between groups, while higher cumulative transfusion volumes at 24 hours are predominantly observed among patients requiring urgent intervention, consistent with ongoing haemorrhage in a subset of patients. Between-group differences were assessed using non-parametric statistical tests.

PRBC = Packed red blood cells
